# Supplementary material for: Transcript Abundance Patterns of 9- and 13-Lipoxygenase Subfamily Gene Members in Response to Abiotic Stresses (Heat, Cold, Drought or Salt) in Tomato (Solanum lycopersicum L.) Highlights Member-Specific Dynamics Relevant to Each Stress
Source: Genes (Basel). 2019 Sep 5;10(9):683. doi: 10.3390/genes10090683 (PMC6771027; doi:10.3390/genes10090683)

Supplementary Information

Transcript Abundance Patterns of 9- and 13-Lipoxygenase Subfamily Gene Members in Response to Abiotic Stresses (Heat, Cold, Drought or Salt) in Tomato (*Solanum lycopersicum* L.) Highlights Member-Specific Dynamics Relevant to Each Stress

Rakesh K. Upadhyay [1,2,*], Avtar K. Handa [2] and Autar K. Mattoo [1,*]

[1] Sustainable Agricultural Systems Laboratory, USDA-ARS, Henry A. Wallace Beltsville Agricultural Research Center, Beltsville, MD 20705-2350, USA

[2] Department of Horticulture and Landscape Architecture, Purdue University, W. Lafayette, IN 47907-2010, USA; ahanda@purdue.edu

* Correspondence: rakesh.upadhyay@usda.gov or rkumarup@purdue.edu (R.K.U.); autar.mattoo@usda.gov (A.K.M.)

Supplementary Tables and Figures

Table S1. List of genes and their primer sequences used for quantitative real– time PCR (qRT-PCR) analysis.

Figure S1. Line Graphs for qRT-PCR analysis of tomato (S. lycopersicum cv. Ailsa Craig) LOX gene family members in response to heat (42ºC) treatment.

Figure S2. Line Graphs for qRT-PCR analysis of tomato (S. lycopersicum cv. Ailsa Craig) LOX gene family members in response to cold (4ºC) treatment.

Figure S3. Line Graphs for qRT-PCR analysis of tomato (S. lycopersicum cv. Ailsa Craig) LOX gene family members in response to drought stress.

Figure S4. Line Graphs for qRT-PCR analysis of tomato (S. lycopersicum cv. Ailsa Craig) LOX gene family members in response to salt treatment.

Table S1: List of genes and their primer sequences used for quantitative real–time PCR (qRT-PCR) analysis.

Gene Name	SGN ID	Forward Primer (5′-3′)	Reverse Primer (5′-3′)
SlTIP41	Solyc10g049850	AACCACATTTCAGGCCTTGTCTT	CATGGAGTTTTTGAGTCTTCTGCAT
SlUBI3	Solyc01g056940	TCGTAAGGAGTGCCCTAATGCTGA	CAATCGCCTCCAGCCTTGTTGTAA
SlLOX1	Solyc08g014000	GATGAGATATACCTCGGACAACGA	GGCCTGTTCTGTTTGTCAATATGT
SlLOX2	Solyc01g099190	GGACTCAATTGAATGGACAAAGGA	CCCTGACCTGTTCTTCCAACTCTT
SlLOX3	Solyc01g006540	AACCTGGAGTTACTGGAAAAGGTG	GCAAAAAGAGCTAGCACACATGAT
SlLOX4	Solyc03g122340	TAGGTGTGGTGCTGGTGTATTACC	CCGCCCTATTTATGGGCTTTAT
SlLOX5	Solyc01g099160	GGGAAAAAGTGAAAGGAACAGTTG	GAAAAGAAACTCTTCTGCCAAGGA
SlLOX6	Solyc09g075860	TTACTATACCCCAATGCCTCAGGT	GACATAGAAGAATGGGTAGGCACA
SlLOX7	Solyc01g099200	GGCCTAAACACCACAAAGATCCTA	ACCACTTCAGATGGATCAAGCTCT
SlLOX8	Solyc08g029000	GCTTTTGAGAGATTTGGGAAGAAG	AGTCCCTGTTCACTTGTAGGGAAG
SlLOX9	Solyc01g099180	GGTGATCATGAGAAATGGAAGAAC	GGAATTCCTTTGCCAGTGAGAC
SlLOX10	Solyc12g011040	TTATTGGGCTGAGGATCCTGTAAT	ATAAGGCATAACACCAGCTCCATT
SlLOX11	Solyc05g014790	TTCCGAACAGCATCTCTATCTGAC	CTTCTCAACAGCTTACAACCCTCA
SlLOX12	Solyc01g006560	CCCTAAGCAAAACTCCACCATCTA	ATGGAATAAGGGTTTTTGTGGATT
SlLOX13	Solyc01g099210	GGACCACAGATGAAGAACCATTAC	CTCTGTTCTTCAAGTTCGGATCAT
SlLOX14	Solyc09g075870	CAAACTCTTATGGGCATCTCATTG	ACCAACTTGTCACTGAATTTCTGC

Figure S1. **Line Graphs for qRT-PCR analysis of tomato (*S. lycopersicum* cv. Ailsa Craig) *LOX* gene family members in response to heat (42⁰C) treatment.** qRT-PCR data of tomato *LOX* genes in response to heat was presented in comparison to non-treated control (0 h) where later was considered as calibrator in qRT-PCR data calculation and used for defining statistical significance of treatment data points where *P<0.05, **P<0.01,***P<0.001 and ****P<0.0001. A minimum of 3 biological replicates, where each biological replicate was comprised of two technical replicates, were used for each time point. *SlTIP41 and SlUBI3* housekeeping genes were used to normalize the expression of target genes.

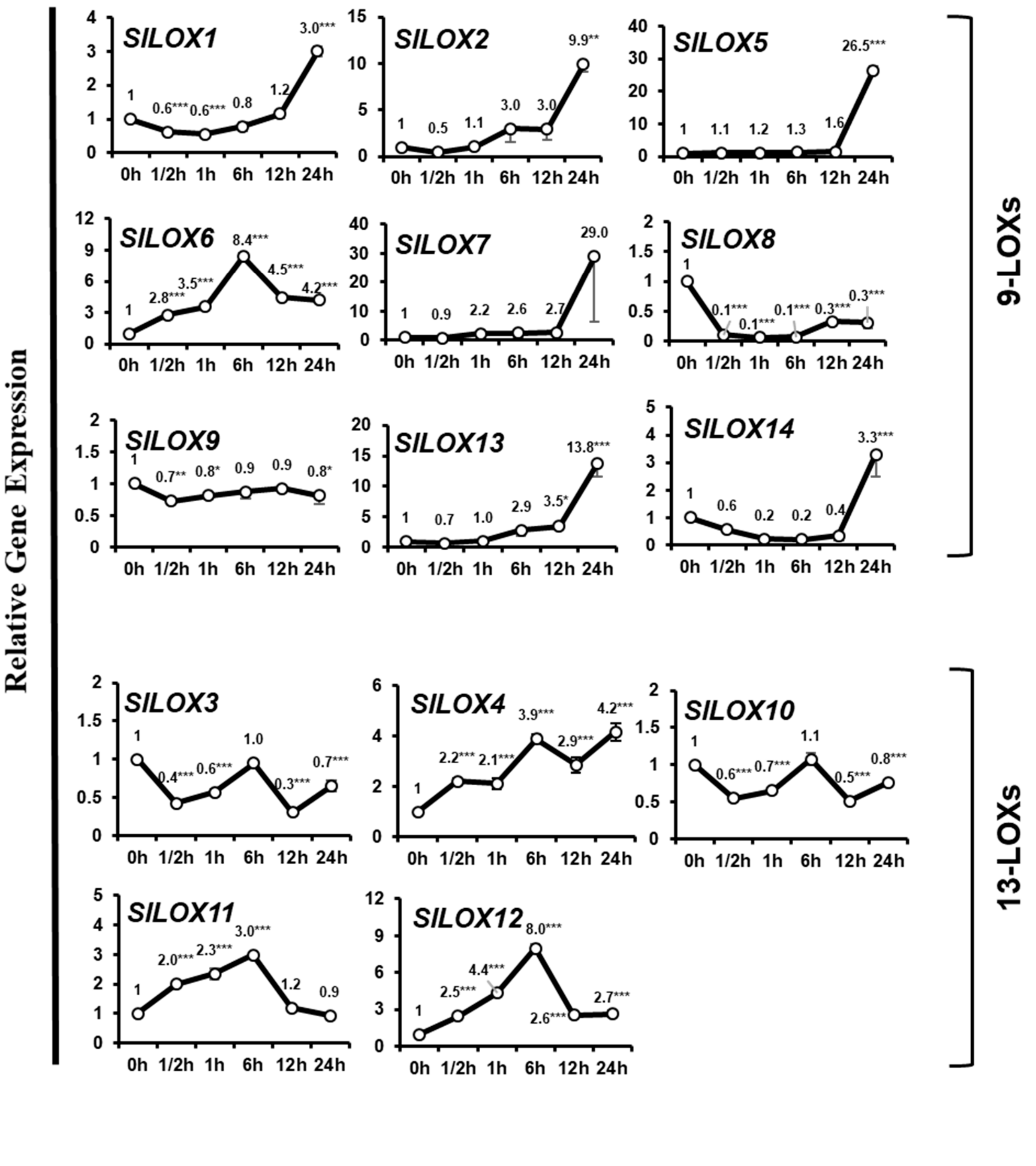

Figure S2. **Line Graphs for qRT-PCR analysis of tomato (*S. lycopersicum* cv. Ailsa Craig) *LOX* gene family members in response to cold (4ºC) treatment.** qRT-PCR data of tomato *LOX* genes in response to cold was presented in comparison to non-treated control (0 h) where later was considered as calibrator in qRT-PCR data calculation and used for defining statistical significance of treatment data points where *P<0.05, **P<0.01,***P<0.001 and ****P<0.0001. A minimum of 3 biological replicates, where each biological replicate was comprised of two technical replicates, were used for each time point. *SlTIP41 and SlUBI3* housekeeping genes were used to normalize the expression of target genes.

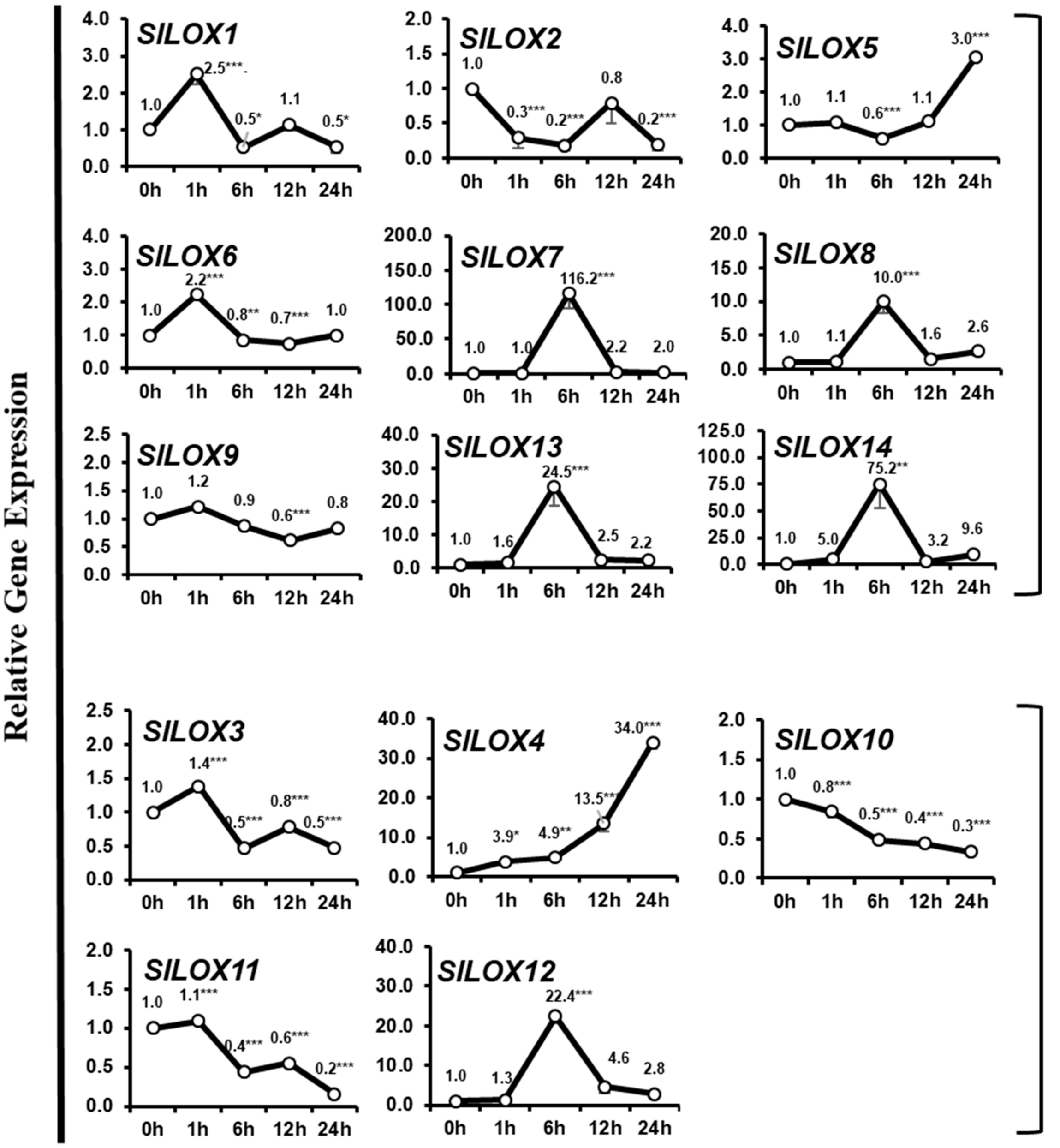

Figure S3. **Line Graphs for qRT-PCR analysis of tomato (*S. lycopersicum* cv. Ailsa Craig) *LOX* gene family members in response to drought stress.** qRT-PCR data of tomato *LOX* genes in response to drought was presented in comparison to non-treated control (0 h) where later was considered as calibrator in qRT-PCR data calculation and used for defining statistical significance of treatment data points where *P<0.05, **P<0.01,***P<0.001 and ****P<0.0001. A minimum of 3 biological replicates, where each biological replicate was comprised of two technical replicates, were used for each time point. *SlTIP41 and SlUBI3* housekeeping genes were used to normalize the expression of target genes.

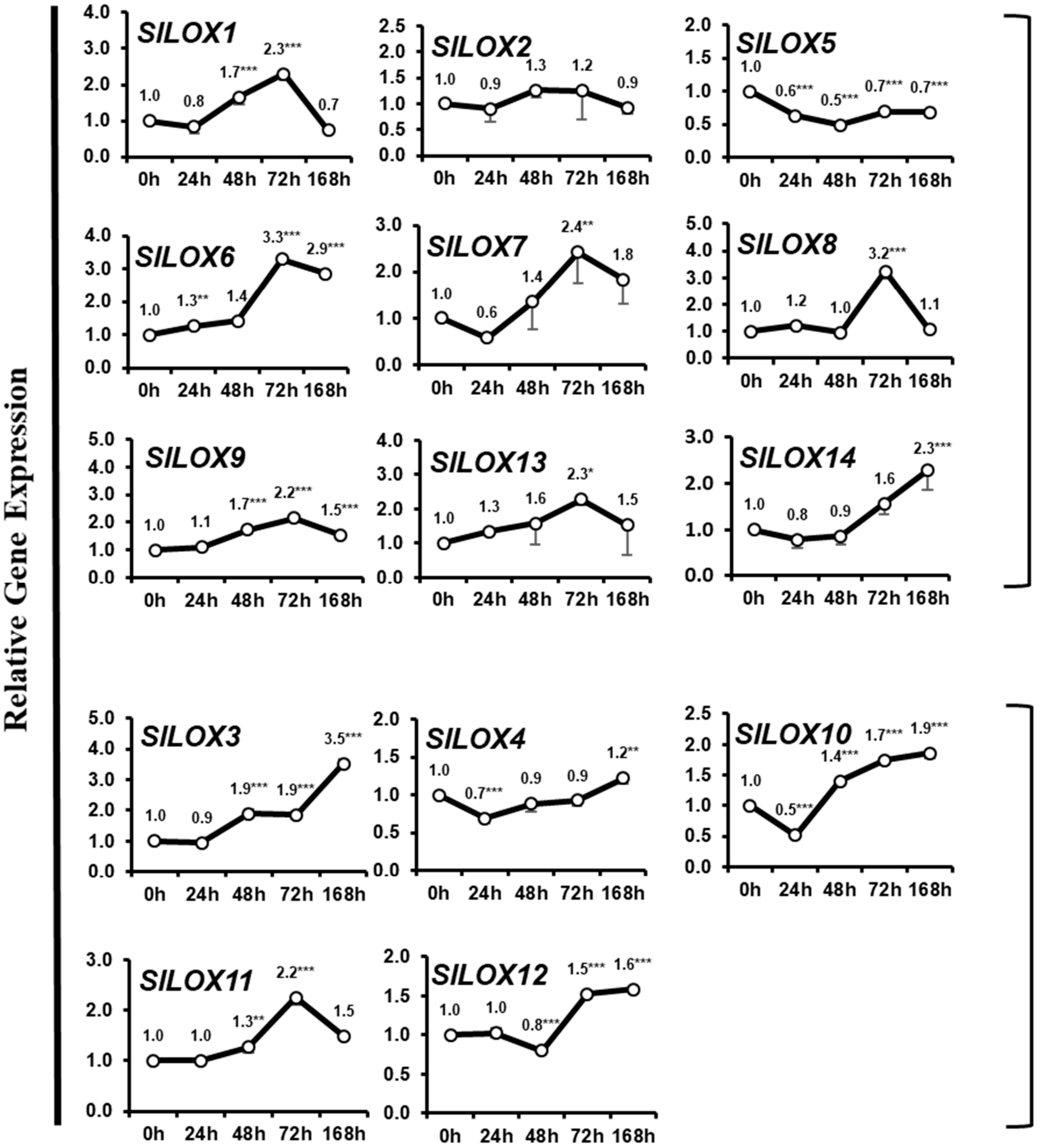

Figure S4. **Line Graphs for qRT-PCR analysis of tomato (*S. lycopersicum* cv. Ailsa Craig) *LOX* gene family members in response to salt treatment.** qRT-PCR data of tomato *LOX* genes in response to salt was presented in comparison to non-treated control (0 h) where later was considered as calibrator in qRT-PCR data calculation and used for defining statistical significance of treatment data points where *P<0.05, **P<0.01,***P<0.001 and ****P<0.0001. A minimum of 3 biological replicates, where each biological replicate was comprised of two technical replicates, were used for each time point. *SlTIP41 and SlUBI3* housekeeping genes were used to normalize the expression of target genes.

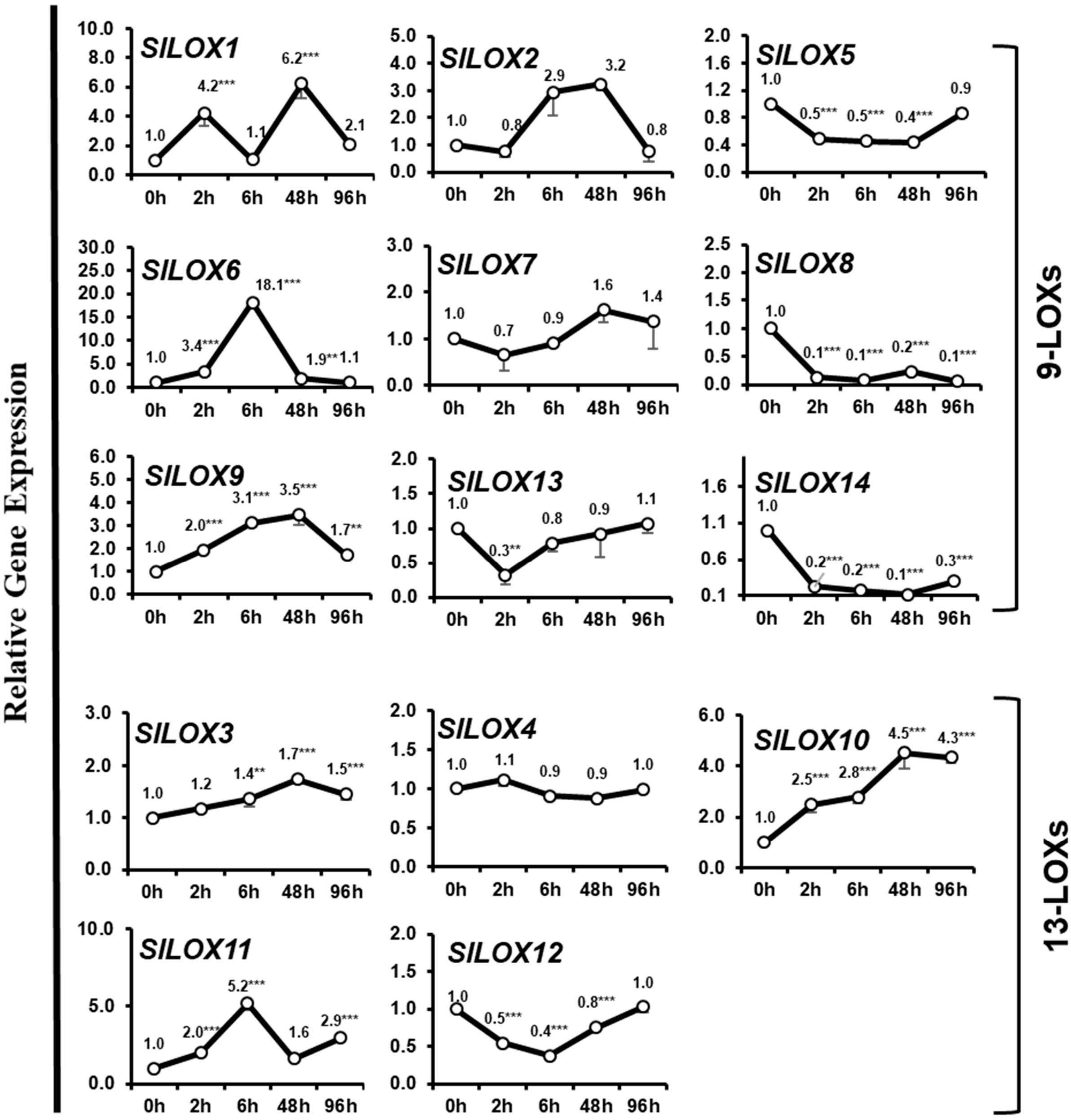

Supplement: Supplementary file 1 [file genes-10-00683-s001.pdf]
